# The contribution of *CHEK2 *to the *TP53*-negative Li-Fraumeni phenotype

**DOI:** 10.1186/1897-4287-7-4

**Published:** 2009-02-17

**Authors:** Marielle WG Ruijs, Annegien Broeks, Fred H Menko, Margreet GEM Ausems, Anja Wagner, Rogier Oldenburg, Hanne Meijers-Heijboer, Laura J van't Veer, Senno Verhoef

**Affiliations:** 1 Family Cancer Clinic, The Netherlands Cancer Institute, Amsterdam, The Netherlands; 2 Department of Experimental Therapy, The Netherlands Cancer Institute, Amsterdam, The Netherlands; 3 Department of Clinical Genetics and Human Genetics, VU University Medical Centre, Amsterdam, The Netherlands; 4 Department of Medical Genetics, University Medical Centre, Utrecht, The Netherlands; 5 Department of Clinical Genetics, Erasmus Medical Centre, Rotterdam, The Netherlands

## Abstract

**Background:**

*CHEK2 *has previously been excluded as a major cause of Li-Fraumeni syndrome (LFS). One particular *CHEK2 *germline mutation, c.1100delC, has been shown to be associated with elevated breast cancer risk. The prevalence of CHEK2*1100delC differs between populations and has been found to be relatively high in the Netherlands. The question remains nevertheless whether *CHEK2 *germline mutations contribute to the Li-Fraumeni phenotype.

**Methods:**

We have screened 65 Dutch *TP53*-negative LFS/LFL candidate patients for *CHEK2 *germline mutations to determine their contribution to the LFS/LFL phenotype.

**Results:**

We identified six index patients with a *CHEK2 *sequence variant, four with the c.1100delC variant and two sequence variants of unknown significance, p.Phe328Ser and c.1096-?_1629+?del.

**Conclusion:**

Our data show that *CHEK2 *is not a major LFS susceptibility gene in the Dutch population. However, *CHEK2 *might be a factor contributing to individual tumour development in *TP53*-negative cancer-prone families.

## Background

Li-Fraumeni syndrome (LFS) is a rare autosomal dominant cancer syndrome predisposing for bone and soft tissue sarcoma, breast cancer, brain tumour, adrenocortical carcinoma and leukaemia [1]. The classical LFS criteria are: a proband with sarcoma aged under 45 years and a first-degree relative with any cancer aged under 45 years, plus a first or second-degree relative in the same lineage with any cancer under the age of 45 years or sarcoma at any age [2]. In addition, Li-Fraumeni-like syndrome (LFL) criteria have been formulated as a proband with any childhood tumour or a sarcoma, brain tumour or adrenocortical tumour diagnosed under 45 years of age and a first or second-degree relative in the same lineage with a typical LFS tumour at any age, plus a first or second-degree relative in the same lineage younger than 60 years with any cancer [3]. Less stringent LFL criteria were formulated by Eeles et al. as two first or second-degree relatives with typical LFS-extended tumours (classical LFS tumours plus melanoma, prostate cancer and pancreatic cancer) at any age [4]. The Chompret criteria for *TP53 *germline mutation testing have been updated in 2008 as: (1) a proband with a tumour belonging to the LFS tumour spectrum (sarcomas, brain tumours, pre-menopausal breast cancer, adrenocortical carcinoma, leukaemia, lung bronchoalveolar cancer) cancer before 46 years of age and at least one first or second-degree relative with an LFS tumour before 56 years of age or multiple tumours; or (2) a proband with multiple tumours two of which belong to the narrow LFS tumour spectrum and the first of which occurred before 46 years of age; or (3) a patient with adrenocortical carcinoma or a patient with breast cancer before 36 years of age without *BRCA *mutation, irrespective of the family history [5].

In 1990 germline mutations in the *TP53 *gene were described in LFS [6]. So far, 419 *TP53*-positive families have been reported (IARC mutation database, R13, November 2008 [7]). At present, in approximately 75% of LFS and 40% of LFL families, a germline *TP53 *mutation can be detected [8]; i.e. 25% to 60% of LFS/LFL families do not carry a detectable germline *TP53 *mutation, implying the existence of alternative LFS susceptibility genes.

CHEK2 is a cell cycle checkpoint kinase involved in DNA repair, cell death and cell cycle control by stabilizing the p53 protein [9]. In 1999 Bell et al. first described the possible association of the *CHEK2 *gene with Li-Fraumeni syndrome [10]. Subsequent studies have addressed the possible contribution of *CHEK2 *germline mutations to LFS and LFL syndrome, but could not confirm *CHEK2 *as a major gene involved in LFS [10-18].

In other studies, the specific *CHEK2 *c.1100delC frameshift mutation was shown to be associated with an elevated breast cancer risk [19-22] and it has been suggested that it contributes to a hereditary breast and colorectal cancer phenotype [23]. The prevalence of this c.1100delC mutation seems to differ according to ethnic backgrounds and populations and is relatively high in the Netherlands [19,24]. We have investigated the *CHEK2 *gene mutation status of 65 index patients from 65 Dutch LFS/LFL families and families suggestive of LFS who had tested negative for *TP53 *germline mutations, to determine the contribution of *CHEK2 *germline mutations to the phenotype in those families.

## Methods

All 65 affected index patients had been assessed and counselled in various clinical genetics centres because of the occurrence of different cancer types related to LFS and had as a consequence been tested for *TP53 *germline mutations. On the basis of the available clinical data, the *TP53*-negative families were classified into 3 groups: 1) classical LFS [2], 2) LFL syndrome according to Birch [3] or Eeles [4] and 3) LFS-suggestive, including childhood onset (under 18 years) sarcoma or brain tumours, two or more primary tumours at any age, two first-degree relatives with a tumour at any age, of which at least one relative has a typical LFS tumour or breast cancer under 30 years of age (without *BRCA1 *or *BRCA2 *mutations) (Table 1). In families with multiple breast cancer cases and individuals with breast and ovarian cancer, *BRCA1 *or *BRCA2 *mutations were excluded, according to standard procedures. Details are available on request.

**Table 1:** Number of *TP53 *negative families available for *CHEK2 *gene analysis divided into 3 groups: LFS, LFL, or LFS-suggestive family history (n = 65), including the cancer type in tested individuals.

	(family) history		Complete *CHEK2 *mutation analysis (n = 34)	1100delC mutation analysis and DNA rearrangements (n = 31)	Cancer type in tested individuals: B/S/other
1	LFS		1	0	1/0/0

2	LFL		20	15	18/7/10

3	LFS-suggestive		13	16	17/5/7

		*-childhood onset sarcoma or brain tumour*	*1*	*1*	*0/1/1*

		*-at least 2 primary tumours*	*3*	*7*	*5/1/4*

		*-2 first degree relatives with cancer*	*5*	*6*	*6/3/2*

		*-breast cancer before 30 years*	*4*	*2*	*6/0/0*

LFS = Li-Fraumeni syndrome

LFL = Li-Fraumeni-like syndrome according to Birch or Eeles

B = breast cancer

S = sarcoma

Other = other cancers, including adrenal cortical tumour, bladder cancer, brain tumour, colon cancer, kidney cancer, leukaemia, lung cancer, melanoma, non-Hodgkin lymphoma, ovarian cancer and thyroid cancer.

DNA from peripheral blood lymphocytes was isolated according to standard procedures. Screening for *TP53 *germline mutations was performed by sequence analysis of all coding exons (2–11) including flanking intron-exon boundaries (details are available on request) and multiplex ligation-dependent probe amplification (MLPA) [25] (*TP53 *MLPA KIT, MRC Holland). In 34 *TP53*-negative LFS, LFL, or LFS-suggestive families all exons and flanking intron-exon boundaries of the *CHEK2 *gene were investigated using denaturing gradient gel electrophoresis (DGGE, see Table 1) [26]. All possible candidate variants, identified as aberrant DGGE fragments, were confirmed by sequence analysis. To avoid amplification of pseudogenes, a long range PCR was performed first for exons 10 to 14, followed by a nested PCR. Data on exons 1–10 were obtained for all patients, on exons 11–14 for 29 of the 34 individuals. All 65 *TP53*-negative individuals were screened for the c.1100delC *CHEK2 *mutation and *CHEK2 *DNA rearrangements by multiplex ligation-dependent probe amplification (MLPA, see Table 1). Details are available on request (*CHEK2 *MLPA KIT, MRC Holland). Mutation analysis was performed using the following reference sequence: CHEK2 (AF086904.1, GI:3982839, ).

Sequence variants were weighted according to their potential pathogenicity. Three silent sequence variants were seen and not further analysed: c.252A>G, p.Glu84Glu in exon 1, a previously reported silent polymorphism [15], found once, c.1566C>T, p.Pro522Pro and c.1608A>G, p.Pro536Pro, both in exon 14, found in five and seven families, respectively. For these three variants two splice site prediction programs were used, NetGene2 Server  and BDGP Splice Site Prediction/Neural Network ; no alternative splice sites were predicted.

When possible, the presence of a sequence variant detected in an index patient was investigated in other affected relatives. A control group of 150 anonymous Dutch (male and female) blood donors was analysed by DGGE to determine the prevalence of the sequence variants in a general population sample.

A chi-square test was used to determine the statistical significance of the proportion of *CHEK2 *mutation carriers in our study group compared to healthy controls.

## Results and discussion

Sixty-five *TP53*-negative individuals from 65 families were screened for the *CHEK2 *1100delC germline mutation and DNA rearrangements. Thirty-four of these individuals were screened comprehensively by DGGE for *CHEK2 *mutations. Six index patients were found to carry a possibly pathogenic germline *CHEK2 *sequence variant.

**Figure 1:**
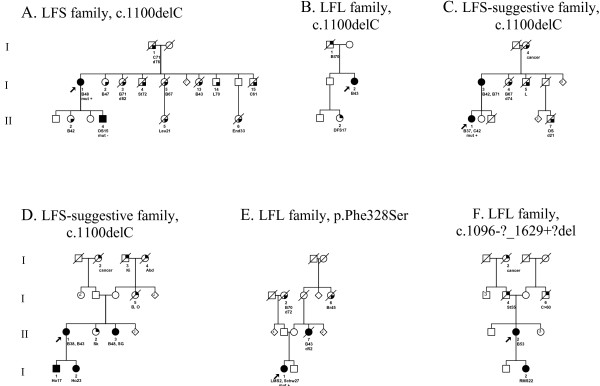
**Pedigrees of germline *CHEK2 *sequence variation families**. Square symbols indicate males, round symbols indicate females, line across symbol means deceased individual. Filled symbols indicate affected individuals with diagnosis confirmed by pathology reports. A quarterly filled symbol indicate affected individuals with diagnosis by family history. Tumour type and age at diagnosis of the tumours are indicated below the individual identifiers, d = age of death. The index patient is indicated with an arrow. Abd = abdominal cancer, B = breast cancer, Bl = bladder cancer, Br = brain tumour, C = colorectal cancer, cancer = cancer of unknown origin, DFS = dermatofibrosarcoma, End = endometrial cancer, Ho = Hodgkin lymphoma, Ki = kidney cancer, L = lung cancer, Leu = leukaemia, LMS = Leiomyosarcoma, O = ovarian cancer, Oes = oesophagus carcinoma, OS = osteosarcoma, RMS = rhabdomyosarcoma, Schw = schwannoma, SG = salivary gland cancer, Sk = skin cancer, St = stomach cancer, mut + = mutation detected, mut - = mutation excluded.

The c.**1100delC in exon 10 of the *CHEK2 *gene**, a mutation located in the kinase domain of the gene and abolishing the kinase activity of the protein, was detected in four index patients. In one family, a classical LFS family, the c.1100delC was detected in a patient who developed breast cancer at the age of 48 years (Figure 1A), which is in line with the c.1100delC acting as a low penetrance breast cancer susceptibility allele [19]. Relatives with a 50% chance of being a c.1100delC carrier in this family who had developed breast cancer were not available for testing. However, it is not likely to be the LFS-causing mutation in this family, considering the absence of the c.1100delC in the patient's son who developed a sarcoma at 15 years of age. In an LFL and LFS-suggestive family, the patients identified as carrying the c.1100delC had breast cancer (Figure 1B and 1D); in a fourth family, a LFS-suggestive family, the patient identified with the c.1100delC sequence variant had both breast and colorectal cancer (Figure 1C). No additional material was available for testing to see if and how the mutation segregates in these families. In all four of the c.1100delC families, this sequence variant seemed to be associated with breast cancer or breast and colorectal cancer, rather than LFS. The reported frequency of the *CHEK *c.1100delC in Dutch controls is 1.4%, in Dutch breast cancer patients not selected for family history 2.5% and in Dutch *BRCA1/2*-negative families with breast cancer 4.9%[19]. In our sample the frequency was 6.2% (4/65), significantly different from that for healthy controls (p = 0.006).

Another sequence variant, c.**983T>C, p.Phe328Ser in exon 8**, localised in the kinase domain of the gene, was detected in a female patient who had developed a leiomyosarcoma at 2 years of age and a schwannoma at 27 years of age (Figure 1E). The family of the index patient fulfilled the LFL criteria (Eeles [4]). The parents of the index patient are both healthy and over 60 years of age. A maternal aunt died of breast cancer at 45 years of age and a sister of the index patient's maternal grandmother died of a brain tumour; no material was available for testing. Under the assumption that these two affected family members were carriers of the mutation, penetrance would be incomplete with non-penetrance exhibited by two older healthy obligate mutation carriers. The hypothesis that the exon 8 mutation caused the complete LFL phenotype in this family is unlikely although a de novo mutation, contributing to the disease in the index patient, cannot be excluded. The p.Phe328Ser missense mutation has not been described in the literature before and was not found in 150 healthy Dutch controls. The phenylalanine in this position is conserved in mice and frogs but not in either zebrafish or C. Elegans (Ensembl, v39-Jun 2006 [27]).

One *CHEK2 *DNA rearrangement was found, **c.1096-?_1629+?del**, a deletion of exons 10–14 of the gene. The family fulfilled the LFL criteria according to Birch[3] and the index patient developed breast cancer at the age of 55 years. A deletion of this size in the kinase domain of the gene will probably abolish the kinase activity. This deletion has not been described in the literature before. A deletion of exon 9–10 predicting protein truncation at codon 381 was discovered as a founder mutation among patients of Czecholovakian ancestry with breast cancer [17]. Unfortunately, no material from the daughter who developed a rhabdomyosarcoma at 22 years of age was available. Four sibs of the index patient were healthy; her father developed stomach cancer at 55 years of age.

So far, 8 studies have been published on *CHEK2 *analysis of a total of 196 *TP53*-negative LFS families or families suggestive of LFS (Table 2) [10-17]. Of the seven variants presented, only the c.1100delC, the p.Ile157Thr and the p.Arg145Trp mutation are of reported functional significance. Bell et al [10] found the p.Ile157Thr in an index patient with three primary cancers; no other family members were tested. Allinen et al. [11] screened the *CHEK2 *gene in 21 LFS/LFL families and only found the p.Ile157Thr mutation; since it was found in healthy controls as well, they concluded that it does not contribute to an LFL-associated breast cancer risk. Some authors found an association between the p.Ile157Thr mutation and risk of female breast cancer [28-30], others found no association [31]. The p.Arg145Trp, leading to a destabilised protein, was described in a Li-Fraumeni-like kindred; it was only tested in one family-member with a sarcoma at 20 years and breast cancer at 42 years. It was not found in 200 controls [13].

**Table 2:** Literature on *CHEK2 *analysis in LFS and LFS-related families

	**number of families tested: LFS/LFL/suggestive**	**total *CHEK2 *analysis**	**CHEK2 1100delC analysis + DNA rearrangements**	**mutations found**
*Allinen et al*. [11]	1/20/0	21	0	p.Ile157Thr

*Bell et al*. [10]	4/18*	22	0	c.1100delC p.Ile157Thr

*Bougeard et al*. [12]	0/4/0	4	0	-

*Lee et al*. [13]	10/49*	59	0	p.Arg145Trp p.Arg3Trp p.Ile157Thr

*Siddiqui et al*. [16]	1/13/1	0	15	-

*Sodha et al*. [15]	5/21/0	26	0	IVS5-11G>A c.483-485delAGA

*Vahteristo et al*. [14]	1/6/32	39	0	c.1100delC

*Walsh et al*. [16]	3/7/0	10		-

our results	1/35/29	34	31	p.Phe328Ser c.1100delC c.1081-?_1771+?del

* = LFL and LFS-suggestive combined, subdivision not further mentioned

In our study six index patients were found to carry a *CHEK2 *sequence variant by screening 65 *TP53*-negative index patients, with no evidence that the sequence variants found caused the complete LFS phenotype in their families. Our data are in line with the hypothesis that the *CHEK2 *c.1100delC might be associated with an elevated breast cancer risk [19,20], and possibly with a breast and colorectal cancer phenotype [23] or more generally a multi-organ cancer susceptibility [32]. We propose that the germline *CHEK2 *sequence variants contribute to tumour development in the index patients. Without these tumours, the families would not have fulfilled the established LFS/LFL criteria and *TP53 *germline mutation testing would not have been indicated. In this way, the individual *CHEK2 *sequence variants may contribute to the Li-Fraumeni phenotype seen in these families.

Because only 75% of classical LFS families and 40% of LFL families have germline *TP53 *mutations, research groups have looked at candidate genes like *Bcl10 *[33], *CDKN2 *[34,35], *TP63 *[12], *PTEN *[34,36], *CHEK1 *[10,14] and *BAX *[37]; no possible alternative LFS genes were found.

Two polymorphisms, p.Arg72Pro (*TP53 *gene) and SNP309 T>G (*MDM2 *gene), have been shown to have a modifying effect, resulting in an earlier age of onset of cancer in *TP53 *mutation carriers [38,39]; there is even a synergistic effect when both polymorphisms are present. These are proposed examples of modifying factors or low penetrance gene mutations that play a role in age of onset and tumour clustering in cancer-prone families [40]. In our present study group, we investigated the possible modifier effects of these polymorphisms but found no association with an earlier age of tumour onset [41] (p.Arg72Pro, data not shown). We did find a larger proportion of homozygotes for the G-allele of *MDM2 *SNP309 in our *TP53*-negative group, suggesting a modifier effect on the *TP53 *negative Li-Fraumeni phenotype.

## Conclusion

Our data illustrate that *CHEK2 *is not a major LFS susceptibility gene in the Dutch population. The *CHEK2 *gene might be a factor contributing to individual tumour development in families that are subsequently recognised as having a Li-Fraumeni phenotype. Although many genes have been excluded as alternative LFS genes, many more modifiers or low penetrance susceptibility genes might occur in families showing a Li-Fraumeni phenotype.

## Competing interests

The authors declare that they have no competing interests.

## References

[B1] Li FP, Fraumeni JF (1969). Soft-tissue sarcomas, breast cancer, and other neoplasms. A familial syndrome?. Ann Intern Med.

[B2] Li FP, Fraumeni JF, Mulvihill JJ, Blattner WA, Dreyfus MG, Tucker MA, Miller RW (1988). A cancer family syndrome in twenty-four kindreds. Cancer Res.

[B3] Birch JM, Hartley AL, Tricker KJ, Prosser J, Condie A, Kelsey AM, Harris M, Jones PH, Binchy A, Crowther D, Craft AW, Eden OB, Evans DGR, Thompson E, Mann JR, Martin J, Mitchell ELD, Santibanez-Koref MF (1994). Prevalence and diversity of constitutional mutations in the p53 gene among 21 Li-Fraumeni families. Cancer Res.

[B4] Eeles RA (1995). Germline mutations in the TP53 gene. Cancer Surv.

[B5] Bougeard G, Sesboue R, Baert-Desurmont S, Vasseur S, Martin C, Tinat J, Brugieres L, Chompret A, de Paillerets BB, Stoppa-Lyonnet D, Bonaiti-Pellie C, Frebourg T (2008). Molecular basis of the Li-Fraumeni syndrome: an update from the French LFS families. J Med Genet.

[B6] Malkin D, Li FP, Strong LC, Fraumeni JF, Nelson CE, Kim DH, Kassel J, Gryka MA, Bischoff FZ, Tainsky MA, Friend SH (1990). Germ line p53 mutations in a familial syndrome of breast cancer, sarcomas, and other neoplasms. Science.

[B7] Petitjean A, Mathe E, Kato S, Ishioka C, Tavtigian SV, Hainaut P, Olivier M (1990). Impact of mutant p53 functional properties on TP53 mutation patterns and tumor phenotype: lessons from recent developments in the IARC TP53 database. Hum Mutat.

[B8] Varley JM (2003). Germline TP53 mutations and Li-Fraumeni syndrome. Hum Mutat.

[B9] Bartek J, Falck J, Lukas J (2001). CHK2 kinase – a busy messenger. Nat Rev Mol Cell Biol.

[B10] Bell DW, Varley JM, Szydlo TE, Kang DH, Wahrer DC, Shannon KE, Lubratovich M, Verselis SJ, Isselbacher KJ, Fraumeni JF, Birch JM, Li FP, Garber JE, Haber DA (1999). Heterozygous germ line hCHK2 mutations in Li-Fraumeni syndrome. Science.

[B11] Allinen M, Huusko P, Mantyniemi S, Launonen V, Winqvist R (2001). Mutation analysis of the CHK2 gene in families with hereditary breast cancer. Br J Cancer.

[B12] Bougeard G, Limacher JM, Martin C, Charbonnier F, Killian A, Delattre O, Longy M, Jonveaux P, Fricker JP, Stoppa-Lyonnet D, Flaman JM, Frebourg T (2001). Detection of 11 germline inactivating TP53 mutations and absence of TP63 and HCHK2 mutations in 17 French families with Li-Fraumeni or Li-Fraumeni-like syndrome. J Med Genet.

[B13] Lee SB, Kim SH, Bell DW, Wahrer DC, Schiripo TA, Jorczak MM, Sgroi DC, Garber JE, Li FP, Nichols KE, Varley JM, Godwin AK, Shannon KM, Harlow E, Haber DA (2001). Destabilization of CHK2 by a missense mutation associated with Li- Fraumeni Syndrome. Cancer Res.

[B14] Vahteristo P, Tamminen A, Karvinen P, Eerola H, Eklund C, Aaltonen LA, Blomqvist C, Aittomaki K, Nevanlinna H (2001). p53, CHK2, and CHK1 genes in Finnish families with Li-Fraumeni syndrome: further evidence of CHK2 in inherited cancer predisposition. Cancer Res.

[B15] Sodha N, Houlston RS, Bullock S, Yuille MA, Chu C, Turner G, Eeles RA (2002). Increasing evidence that germline mutations in CHEK2 do not cause Li- Fraumeni syndrome. Hum Mutat.

[B16] Siddiqui R, Onel K, Facio F, Nafa K, Diaz LR, Kauff N, Huang H, Robson M, Ellis N, Offit K (2005). The TP53 mutational spectrum and frequency of CHEK2*1100delC in Li-Fraumeni-like kindreds. Fam Cancer.

[B17] Walsh T, Casadei S, Coats KH, Swisher E, Stray SM, Higgins J, Roach KC, Mandell J, Lee MK, Ciernikova S, Foretova L, Soucek P, King MC (2006). Spectrum of mutations in BRCA1, BRCA2, CHEK2, and TP53 in families at high risk of breast cancer. JAMA.

[B18] Evans DG, Birch JM, Narod SA (2008). Is CHEK2 a cause of the Li-Fraumeni syndrome?. J Med Genet.

[B19] Meijers-Heijboer H, Ouweland A van den, Klijn J, Wasielewski M, de Snoo A, Oldenburg R, Hollestelle A, Houben M, Crepin E, Veghel-Plandsoen M, Elstrodt F, van Duijn C, Bartels C, Meijers C, Schutte M, McGuffog L, Thompson D, Easton D, Sodha N, Seal S, Barfoot R, Mangion J, Chang-Claude J, Eccles D, Eeles R, Evans DG, Houlston R, Murday V, Narod S, Peretz T, Peto J, Phelan C, Zhang HX, Szabo C, Devilee P, Goldgar D, Futreal PA, Nathanson KL, Weber B, Rahman N, Stratton MR (2002). Low-penetrance susceptibility to breast cancer due to CHEK2(*)1100delC in noncarriers of BRCA1 or BRCA2 mutations. Nat Genet.

[B20] Vahteristo P, Bartkova J, Eerola H, Syrjakoski K, Ojala S, Kilpivaara O, Tamminen A, Kononen J, Aittomaki K, Heikkila P, Holli K, Blomqvist C, Bartek J, Kallioniemi OP, Nevanlinna H (2002). A CHEK2 genetic variant contributing to a substantial fraction of familial breast cancer. Am J Hum Genet.

[B21] Weischer M, Bojesen SE, Ellervik C, Tybjaerg-Hansen A, Nordestgaard BG (2008). CHEK2*1100delC genotyping for clinical assessment of breast cancer risk: meta-analyses of 26,000 patient cases and 27,000 controls. J Clin Oncol.

[B22] Offit K, Garber JE (2008). Time to check CHEK2 in families with breast cancer?. J Clin Oncol.

[B23] Meijers-Heijboer H, Wijnen J, Vasen H, Wasielewski M, Wagner A, Hollestelle A, Elstrodt F, Bos R Van Den, de Snoo A, Tjon AF, Brekelmans C, Jagmohan S, Franken P, Verkuijlen P, Ouweland A van den, Chapman P, Tops C, Moslein G, Burn J, Lynch H, Klijn J, Fodde R, Schutte M (2003). The CHEK2 1100delC Mutation Identifies Families with a Hereditary Breast and Colorectal Cancer Phenotype. Am J Hum Genet.

[B24] Osorio A, Rodriguez-Lopez R, Diez O, de la Hoya M, Ignacio Martinez J, Vega A, Esteban-Cardenosa E, Alonso C, Caldes T, Benitez J (2004). The breast cancer low-penetrance allele 1100delC in the CHEK2 gene is not present in Spanish familial breast cancer population. Int J Cancer.

[B25] Hogervorst FBL, Nederlof PM, Gille JJP, McElgunn CJ, Grippeling M, Pruntel R, Regnerus R, van Welsem T, van Spaendonk R, Menko FH, Kluijt I, Dommering C, Verhoef S, Schouten JP, van't Veer LJ, Pals G (2003). Large Genomic Deletions and Duplications in the BRCA1 Gene Identified by a Novel Quantitative Method. Cancer Research.

[B26] Broeks A, de Witte L, Nooijen A, Huseinovic A, Klijn JG, van Leeuwen FE, Russell NS, van't Veer LJ (2004). Excess risk for contralateral breast cancer in CHEK2*1100delC germline mutation carriers. Breast Cancer Res Treat.

[B27] Hubbard T, Andrews D, Caccamo M, Cameron G, Chen Y, Clamp M, Clarke L, Coates G, Cox T, Cunningham F, Curwen V, Cutts T, Down T, Durbin R, Fernandez-Suarez XM, Gilbert J, Hammond M, Herrero J, Hotz H, Howe K, Iyer V, Jekosch K, Kahari A, Kasprzyk A, Keefe D, Keenan S, Kokocinsci F, London D, Longden I, McVicker G, Melsopp C, Meidl P, Potter S, Proctor G, Rae M, Rios D, Schuster M, Searle S, Severin J, Slater G, Smedley D, Smith J, Spooner W, Stabenau A, Stalker J, Storey R, Trevanion S, Ureta-Vidal A, Vogel J, White S, Woodwark C, Birney E (2005). Ensembl 2005. Nucleic Acids Res.

[B28] Bogdanova N, Enssen-Dubrowinskaja N, Feshchenko S, Lazjuk GI, Rogov YI, Dammann O, Bremer M, Karstens JH, Sohn C, Dork T (2005). Association of two mutations in the CHEK2 gene with breast cancer. Int J Cancer.

[B29] Kilpivaara O, Vahteristo P, Falck J, Syrjakoski K, Eerola H, Easton D, Bartkova J, Lukas J, Heikkila P, Aittomaki K, Holli K, Blomqvist C, Kallioniemi OP, Bartek J, Nevanlinna H (2004). CHEK2 variant I157T may be associated with increased breast cancer risk. Int J Cancer.

[B30] Gorski B, Cybulski C, Huzarski T, Byrski T, Gronwald J, Jakubowska A, Stawicka M, Gozdecka-Grodecka S, Szwiec M, Urbanski K, Mitus J, Marczyk E, Dziuba J, Wandzel P, Surdyka D, Haus O, Janiszewska H, Debniak T, Toloczko-Grabarek A, Medrek K, Masojc B, Mierzejewski M, Kowalska E, Narod SA, Lubinski J (2005). Breast cancer predisposing alleles in Poland. Breast Cancer Res Treat.

[B31] Dufault MR, Betz B, Wappenschmidt B, Hofmann W, Bandick K, Golla A, Pietschmann A, Nestle-Kramling C, Rhiem K, Huttner C, von LC, Dall P, Kiechle M, Untch M, Jonat W, Meindl A, Scherneck S, Niederacher D, Schmutzler RK, Arnold N (2004). Limited relevance of the CHEK2 gene in hereditary breast cancer. Int J Cancer.

[B32] Cybulski C, Gorski B, Huzarski T, Masojc B, Mierzejewski M, Debniak T, Teodorczyk U, Byrski T, Gronwald J, Matyjasik J, Zlowocka E, Lenner M, Grabowska E, Nej K, Castaneda J, Medrek K, Szymanska A, Szymanska J, Kurzawski G, Suchy J, Oszurek O, Witek A, Narod SA, Lubinski J (2004). CHEK2 is a multiorgan cancer susceptibility gene. Am J Hum Genet.

[B33] Stone JG, Eeles RA, Sodha N, Murday V, Sheriden E, Houlston RS (1999). Analysis of Li-Fraumeni syndrome and Li-Fraumeni-like families for germline mutations in Bcl10. Cancer Lett.

[B34] Burt EC, McGown G, Thorncroft M, James LA, Birch JM, Varley JM (1999). Exclusion of the genes CDKN2 and PTEN as causative gene defects in Li- Fraumeni syndrome. Br J Cancer.

[B35] Portwine C, Lees J, Verselis S, Li FP, Malkin D (2000). Absence of germline p16(INK4a) alterations in p53 wild type Li-Fraumeni syndrome families. J Med Genet.

[B36] Brown LT, Sexsmith E, Malkin D (2000). Identification of a novel PTEN intronic deletion in Li-Fraumeni syndrome and its effect on RNA processing. Cancer Genet Cytogenet.

[B37] Barlow JW, Mous M, Wiley JC, Varley JM, Lozano G, Strong LC, Malkin D (2004). Germ line BAX alterations are infrequent in Li-Fraumeni syndrome. Cancer Epidemiol Biomarkers Prev.

[B38] Bond GL, Hu W, Bond EE, Robins H, Lutzker SG, Arva NC, Bargonetti J, Bartel F, Taubert H, Wuerl P, Onel K, Yip L, Hwang SJ, Strong LC, Lozano G, Levine AJ (2004). A single nucleotide polymorphism in the MDM2 promoter attenuates the p53 tumor suppressor pathway and accelerates tumor formation in humans. Cell.

[B39] Bougeard G, Baert-Desurmont S, Tournier I, Vasseur S, Martin C, Brugieres L, Chompret A, Bressac-de Paillerets B, Stoppa-Lyonnet D, Bonaiti-Pellie C, Frebourg T (2005). Impact of the MDM2 SNP309 and TP53 Arg72Pro polymorphism on age of tumour onset in Li-Fraumeni syndrome. J Med Genet.

[B40] Tabori U, Malkin D (2008). Risk stratification in cancer predisposition syndromes: lessons learned from novel molecular developments in Li-Fraumeni syndrome. Cancer Res.

[B41] Ruijs MW, Schmidt MK, Nevanlinna H, Tommiska J, Aittomaki K, Pruntel R, Verhoef S, Van't Veer LJ (2007). The single-nucleotide polymorphism 309 in the MDM2 gene contributes to the Li-Fraumeni syndrome and related phenotypes. Eur J Hum Genet.

